# Metagenomic Analysis of Bacterial, Archaeal and Fungal Diversity in Two-Stage Anaerobic Biodegradation for Production of Hydrogen and Methane from Corn Steep Liquor

**DOI:** 10.3390/microorganisms11051263

**Published:** 2023-05-11

**Authors:** Galina Stoyancheva, Lyudmila Kabaivanova, Venelin Hubenov, Elena Chorukova

**Affiliations:** 1 Department of General Microbiology, The Stephan Angeloff Institute of Microbiology, Bulgarian Academy of Sciences, Acad. G. Bonchev Str., bl. 26, 1113 Sofia, Bulgaria; 2 Department of Biotechnology, The Stephan Angeloff Institute of Microbiology, Bulgarian Academy of Sciences, Acad. G. Bonchev Str., bl. 26, 1113 Sofia, Bulgariaelena_chorukova@yahoo.com (E.C.); 3 Department of Bioinformatics and Mathematical Modelling, Institute of Biophysics and Biomedical Engineering, Bulgarian Academy of Sciences, Acad. G. Bonchev Str., bl. 105, 1113 Sofia, Bulgaria

**Keywords:** metagenomics, waste utilization, green energy production

## Abstract

The main purpose of this study was to identify the microbial communities (bacterial, archaeal and fungal) in a two-stage system of anaerobic bioreactors for the production of hydrogen and methane from the waste substrate—corn steep liquor. Wastes from the food industry are valuable resources with potential in biotechnological production because of their high organic matter contents. In addition, the production of hydrogen and methane, volatile fatty acids, reducing sugars and cellulose content was monitored. Two-stage anaerobic biodegradation processes were performed by microbial populations in the first hydrogen generating bioreactor (working volume of 3 dm^3^) and in the second methane-generating reactor (working volume of 15 dm^3^). Cumulative hydrogen yield reached 2000 cm^3^ or 670 cm^3^/L a day, while the methane production reached a maximum quantity of 3300 cm^3^ or 220 cm^3^/L a day. Microbial consortia in anaerobic digestion systems play an essential role for process optimization and biofuel production enhancement. The obtained results showed the possibility of conducting two separate processes—the hydrogenic (hydrolysis and acidogenesis) and methanogenic (acetogenesis and methanogenesis)—as two stages of anaerobic digestion to favor energy production under controlled conditions with corn steep liquor. The diversity of microorganisms as main participants in the processes in the bioreactors of the two-stage system was followed using metagenome sequencing and bioinformatics analysis. The obtained metagenomic data showed that the most abundant phylum in both bacterial communities was *Firmicutes*—58.61% and 36.49% in bioreactors 1 and 2, respectively. Phylum *Actinobacteria* were found in significant quantities (22.91%) in the microbial community in Bioreactor 1, whereas in Bioreactor 2, they were 2.1%. *Bacteroidetes* are present in both bioreactors. Phylum *Euryarchaeota* made up 0.4% of the contents in the first bioreactor and 11.4% in the second. As the dominant genera among methanogenic archaea are *Methanothrix* (8.03%) and *Methanosarcina* (3.39%), the main fungal representatives were *Saccharomyces cerevisiae*. New knowledge of anaerobic digestion mediated by novel microbial consortia could be widely used to convert different wastes to green energy.

## 1. Introduction

The limitation and depletion of fossil fuel resources make biohydrogen and biomethane worthy alternatives for the future. The recycling of various waste materials from industrial productions is essential in the circular economy, which is nowadays an integral part of sustainability [1]. Anaerobic digestion assists in utilizing wastes and coping with their accumulation while realizing the production of renewable energy carriers. This is why a wide range of wastes obtained from the agricultural and food processing industries as a source of easily degradable organic material is subjected to anaerobic degradation. The production of biogas using food and agricultural waste is related to solving several global problems—such as environmental sustainability; the biogas produced can be used as a source of renewable energy, reducing the need for fossil fuels and resources recovery—involving wastes for biogas production for energy, nutrients and recovery, reducing the waste stream [2]. In the context of climate change, the generation of biogas as a renewable clean energy form has become popular and has been intensively examined in recent decades. Producing sustainable hydrogen and methane production from a definite substrate present major challenges. A two-stage anaerobic digestion process has been suggested as an option to maximize the amount of energy recoverable from biodegradable organic waste, in terms of hydrogen and methane, by enhanced hydrolysis with subsequently higher CH_4_ production. Hydrogen (H_2_) is meant as the fuel of the future, a clean energy carrier that possesses a high energy density and the highest calorific value among other fuels. Moreover, its combustion does not lead to carbon emissions. H_2_ can be produced from available renewable sources in a process called dark fermentation [3]. Methane (CH_4_) is a source of energy that can be used for heat and power production but can also be applied as a gaseous vehicle fuel, in this way replacing natural gas [4]. 

Various substrates had been subjected to anaerobic digestion in view of biogas production. Sole substrates, as well as different mixtures of substrates, could participate in this process and impose their influence on the performance of the steps in digestion and increase its efficiency. Corn steep liquor (CSL) is a byproduct of the corn wet milling industry and is used as a raw material in several industries. CSL is often used as a supplement for microorganism growth in the fermentation processes for the production of various products such as biofuels, enzymes and organic acids. This accessible raw material and waste product from corn starch production is being generated in huge amounts, which causes serious environmental problems. It is often an environmental pollutant and there are some initiatives concerning its incorporation into various biotechnological productions—namely, fructo-oligosaccharide production, natamycin production and lipid and docosahexaenoic acid biosynthesis—as an additive in the fermentation of fresh rice straw silage and others [5,6,7,8].

Anaerobic degradation involves four processes: hydrolysis, acidogenesis, acetogenesis and methanogenesis. Hydrolysis and acidogenesis are rapid processes that release hydrogen and involve many different bacterial species. Acetogenesis and methanogenesis are slower processes that produce methane [9,10]. Anaerobic digestion has appeared to be one of the most reliable processes with a broad future potential [11]. Anaerobic digestion comprises sequential processes accomplished by microorganisms that break down biodegradable material in the absence of oxygen [12]. This unique process is used for industrial or domestic purposes, in many cases to manage waste and/or to produce biofuels [13]. Different studies have been conducted to improve the parameters of anaerobic digestion processes and digester stability with the purpose of increasing biomethane yield [14].

Together with the substrate’s characteristics, no less important is the diversity of microbial communities involved in the production of biogas/methane and biohydrogen. Due to the high complexity of the microbiome in anaerobic biodegradation, it is critically important to investigate the involved microorganisms in depth. The role of the mixed microbial communities participating with their interactions in these processes is an essential point [15]. 

Recently, more and more scientific studies have been reported which examine the composition of microbial communities involved in various industries or are related to processes in the environment and human health [16]. Next-generation sequencing (NGS) technologies are affordable, reliable and have high data throughput; they have nowadays replaced classical culture-based microbiological methods in the characterization of microorganisms, which, in addition to traditional methods, provide strict details about the microbial communities involved. NGS is widely used in the assessment of the structure and function of microbial communities. Metagenomic analysis provides functional insights into the diversity determination by the study of genetic material recovered directly from a definite sample [17].

The process of anaerobic digestion is well studied, but the composition of the microbial communities that carry out these processes is still incompletely elucidated. Obtaining new data on the diversity and structure of the microbial communities in the hydrogen-generating and methane-producing bioreactors was the main objective of this study. Thus, comprehensive information on digesters’ stability and performance for effective energy production can be provided.

## 2. Materials and Methods

### 2.1. Two Stage Bioreactor System

**Figure 1:**
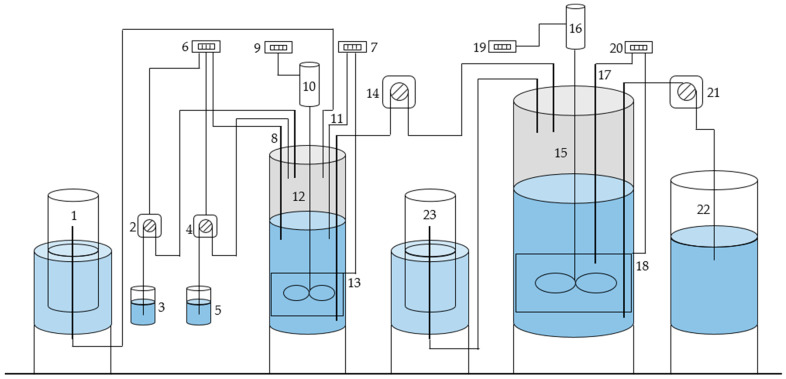
Scheme of the experimental setup. 1. Gasholder for hydrogen; 2. Peristaltic pump for acid; 3. Tank with acid; 4. Peristaltic pump for base; 5. Tank with base; 6. pH-regulator; 7. Temperature regulator of the hydrogen bioreactor; 8. pH-electrode; 9. Speed regulator of the electric motor of the hydrogen bioreactor; 10. Electric motor of the stirrer of the hydrogen bioreactor; 11. Temperature sensor—thermistor Pt-100 of the hydrogen bioreactor; 12. Hydrogen bioreactor—3 L; 13. Heating sleeve of the hydrogen bioreactor; 14. Peristaltic pump for feeding; 15. Methane bioreactor—15 L; 16. Electric motor of the stirrer of the methane bioreactor; 17. Temperature sensor—thermistor Pt-100 of the methane bioreactor; 18. Heating sleeve of the methane bioreactor; 19. Speed regulator of the electric motor of the methane bioreactor; 20. Temperature regulator of the methane bioreactor; 21. Peristaltic pump for outflow of the methane bioreactor; 22. Vessel for outflow of the methane bioreactor; 23. Gasholder for methane.

The two bioreactors were operating with daily feeding mode and the agitator runs continuously. The working volumes of the bioreactors were 3 dm^3^ and 15 dm^3^ (Figure 1). The two bioreactors were equipped with peristaltic pumps for feeding and for effluent subtracting as well as with a system for monitoring and controlling the temperature, estimated at 35 °C ± 0.5 °C for both bioreactors. A pH electrode and a pH regulator, connected with two other peristaltic pumps for the addition of sodium hydroxide or hydrochloric acid solutions, were used for maintaining pH 5.5 at the first bioreactor. Every day, a portion of 2700 cm^3^ liquid from the second bioreactor was withdrawn and an equivalent portion of liquid from the first bioreactor is added. A total 75 g CSL was measured and diluted with distilled water until the total volume reached 2700 cm^3^. This mixture was further added to the first bioreactor for the dark fermentation process with hydrogen production realization. The first bioreactor was maintained at dilution rate D = 0.9 day^−1^, and the second bioreactor at dilution rate D = 0.18 day^−1^.

### 2.2. Substrate

CSL (ADM^®^ Corn Steep Liquor 104) from the manufacturer ADM Razgrad EAD, Razgrad, Bulgaria [18] was used as a raw material for feeding the first bioreactor. The main characteristics of the feed material are presented in Table 1. At the same time, the effluent from the first bioreactor served as an influent/feeding flow for the second (methanogenic) bioreactor (Figure 1).

### 2.3. Inoculum

As an inoculum for the two-phase anaerobic digestion system, the liquid phase from working bioreactor for biogas production was used. Thermal pretreatment was applied for the inactivation of methanogenic microorganisms when the inoculum was intended for the first phase (for hydrogen production). An aliquot of liquid phase was first sieved for not-degraded large particles biomass removal, followed by centrifugation at 4500 rpm. The microbial cells collected in the sediment were re-suspended and washed twice in physiological solution (0.9% NaCl). The microbial cells suspension was heated at 75 °C and, after, it was cooled to room temperature. Then, it was applied in a quantity of 10–30% (*v*/*v*) as an inoculum in the first bioreactor [19]. For the second bioreactor from the two-phase system (methanogenic), the same liquid phase from a working biogas bioreactor was used for starting the process without any pretreatment as it was introduced in the full working volume of the bioreactor (15 dm^3^), and glucose was added for checking and proving of methanogenic activity.

### 2.4. Analytical Methods

The reducing sugars content was analyzed using a method based on the redox reaction between reducing sugars and sodium dinitrosalicylate, giving a reddish-brown derivative. Absorption was measured at a wavelength of 530 nm [20]. Protein content was analyzed according to the Bradford method [21]. The method is based on the reaction of Coomassie Brilliant Blue G-250 with proteins resulting in a shift in the dye color to blue with maximum absorption at 595 nm. Cellulose was determined by the spectrophotometric method, where cellulose-containing materials are released from impurities such as lignin, hemicellulose, xylosans and other low molecular weight compounds by extraction with an acetic–nitric reagent. The purified cellulose was dissolved in 67% H_2_SO_4_, followed by a color reaction with an anthrone reagent [22]. The cellulose concentration was determined after measuring the absorbance at 620 nm. Determination of total solids (TS) and volatile solids (VS) was performed by a standard method by drying a certain volume of sample to a constant weight at 105 ± 3 °C (for determining TS) and subsequent annealing at 575 ± 25 °C (for determining the ash content). The difference between TS and ash content shows the amount of VS in the sample [23]. Because of the high organic content (and to avoid some losses during the annealing), the samples were heated slowly to ensure slow oxidation of organics and to prevent the sample from igniting. The heating, annealing and cooling down to room temperature (in a desiccator) were repeated to reach a constant weight in two consistent measurements. The concentration of volatile fatty acids was determined by a Thermo Scientific gas chromatograph (Focus GC model) equipped with a Split/Splitless injector, column: TG-WAXMS A, (length 30 m, diameter 0.25 mm, film thickness 0.25 µm) and flame ionization detector (FID). Prior to injection, the pH of the sample taken from the bioreactor was adjusted to pH 2.0 with 37% H_3_PO_4_. After one hour, the sample was centrifuged at 15,000 rpm for 10 min and a liquor of the supernatant was mixed with an equal volume of 1% 2.2-dimethyl-butyric acid (as an internal standard). During each run of the chromatographic analysis, the temperature of the oven started from 110 °C and was set to increase to 210 °C and held for 2 min. The FID temperature was set at 210 °C. Released gas volume from both bioreactors was measured using a graduated cylinder in the gas holder working on a water displacement principle. Concentration of hydrogen and methane was measured with gas analyzers, respectively: Dräger X-am7000 (Dräger, Drägerwerk AG & Co. KGaA-Germany, Lübeck, Germany) was used for estimation of CH_4_ and CO_2_ in % by volume (with infrared sensors), and “Gasboard 3100P” (Cubic Sensor and Instrument Co., Ltd., Wuhan, China), equipped with two infrared sensors, was used for measuring the H_2_ and CO_2_ content (in % by volume).

### 2.5. Sample Collection and DNA Extraction

Samples from both bioreactors were collected in sterile vials; then, the samples were stored at −20 °C until further processing. Genomic DNA was extracted using the GeneMATRIX Environmental DNA and RNA Purification Kit (EURx Ltd., Gdańsk, Poland). DNA quantification was conducted using a NanoDrop 1000 spectrophotometer (Thermo Fisher Scientific, Waltham, MA, USA). Sample “Bioreactor_1” was with concentration 50 ng/µL and V = 220 µL, Sample “Bioreactor_2” was with concentration 45 ng/µL and V = 200 µL. Finally, the purified genomic DNA was stored in EB at −20 °C until the next step.

### 2.6. Metagenome Sequencing and Bioinformatics Analysis

Metagenome Amplicon Sequencing is a DNA sequencing method that focuses on sequencing specific target regions. Construction and sequencing of the metagenomic libraries was conducted by Macrogen Inc. (Seoul, Korea). Bacterial metagenomic libraries were prepared using primers targeting the 16S rRNA V3-V4 region (341F-805R). Archaeal metagenomic libraries were constructed using Archaea 16S rRNA specific pr. primers (21F-516R). Fungal metagenomic libraries were constructed with primers for the ITS1-ITS2 region. The six libraries were created with the Herculase II Fusion DNA Polymerase Nextera XT Index V2 Kit. Sequencing was performed with the Sequencing Platform MiSeq Illumina—300 PE (100K reads per sample). The quantity of DNA in all samples was evaluated by picogreen method using Victor 3 fluorometry, followed by Library Size Check and Library Quantity Check. OTU Analysis was performed.

## 3. Results and Discussion

Anaerobic digestion includes the process of wastes transformation into biogas by the aid of methane-producing microorganisms. A two-stage system, producing hydrogen in the first bioreactor, followed by methane production in the second bioreactor, utilizing the waste product—corn steep liquor—was created. Separating the processes into different bioreactors leads to a stable two-stage or even three-stage system to reach significantly higher energy yields [24,25]. 

**Figure 2:**
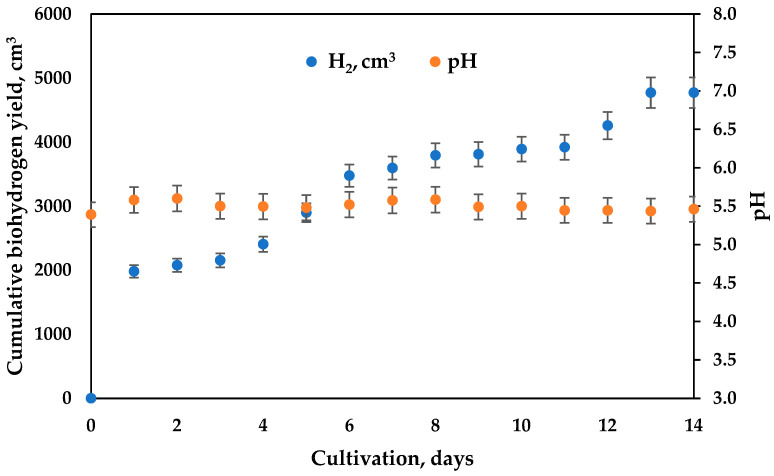
Cumulative hydrogen production from first bioreactor (D = 0.9 day^−1^).

**Figure 3:**
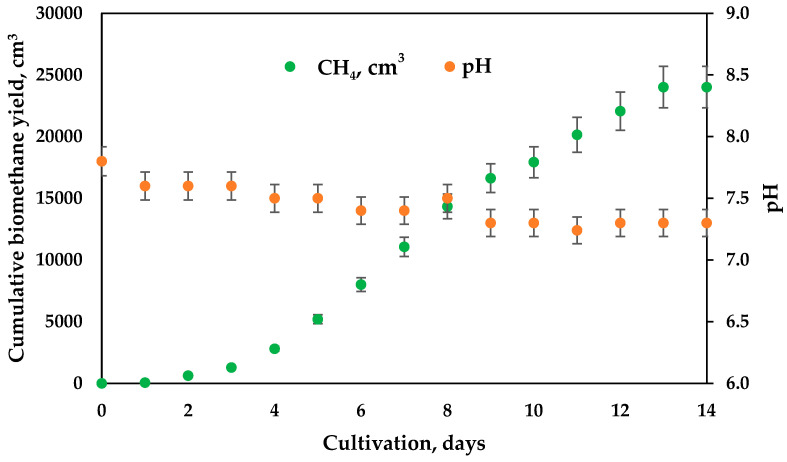
Methane production from second bioreactor and the dynamics in pH value.

Taking into account our previous experience, the pH value in the first bioreactor was maintained at about 5.5 (Figure 2). Because of the high rate of hydrolysis and acidogenesis, which are rapid processes that release organic acids, the automation pH control system is of crucial significance when they are conducted in separate bioreactors. In our case, the hydrogen process was conducted as a process with daily feeding, and the dilution rate (D) was 0.9 day^−1^. In the second bioreactor, where slower processes such as acetogenesis and methanogenesis processes for methane production [9,10] take place, the automation control of pH was not applied. However, pH measurements showed stable values, suitable for the methanogenic process at D = 0.2 day^−1^ (Figure 3). Different anaerobic systems have been proven feasible for the production of biohydrogen. One of our considerations was to synchronize the two bioreactors to operate at dilution rates that allow the effluent from the first bioreactor (F_1_ out) to become influent flow (F_2_ in) to the second bioreactor without the need of an intermediate vessel. Because the optimal conditions for the growth and development of acidogenic (optimal pH 5.0–5.5) and methanogenic (optimal pH 6.5–8.5) microorganisms are different, some authors often observed the inhibition of methanogens in the first bioreactor [26]. Important intermediate products of this process include volatile fatty acids formation. They are then reduced to methane and carbon dioxide [27]. In order to perform the process efficiently, the pH should be maintained—lower than 6.5 and higher than 8.5 in the second step—and be precisely controlled in the first hydrogen-generating step. Some studies have implied that the rate of methanogenesis would be reduced by 57% if the pH value was lower than 6.3 or higher than 7.8 and have even concluded that methanogens could not survive when pH was much outside of the narrower range 7–8 [28]. This important feature (pH) can be largely affected by different byproducts within the biogas formation process. Decreases in alkalinity may be due to the accumulation of organic acid intermediates, often due to the presence of wastes that reduce the ability of methanogens to transform these wastes into biogas. Alkaline levels are closely related to the release of amino acids, chemically turned into ammonia and ammonium ions, while bicarbonates are primary responsible for the buffering capacity and the balancing of the pH of the medium. Sugars decompose into acetate and acetic acid. This is the reason for the drop in the pH values. 

The cumulative hydrogen yield is presented in Figure 2. The maximal hydrogen yield reached 2000 cm^3^ or 670 cm^3^/L a day.

In general, when a single substrate is used in anaerobic digestion, very little hydrogen can be generated. In the study of Longoria et al. [29], they tried to increase the yield of the hydrogen produced with swine manure and other co-substrates, stating that the yield can vary depending on the biodegradability and complexity of the mixture of substrates.

The cumulative biogas/methane yield from the second bioreactor is shown in Figure 3. The maximal quantity obtained was 3300 cm^3^ or 220 cm^3^/L a day.

The obtained results showed the possibility of conducting two separate processes—the hydrogenic hydrolysis and acidogenesis; and methanogenic acetogenesis and methanogenesis—as two stages of anaerobic digestion to favor energy production under controlled conditions with the waste substrate CSL. 

Comparative studies of methane production between single-stage anaerobic digestion systems and two-stage anaerobic digestion systems from agricultural or other residues have revealed that the two-stage process is more attractive in terms of energy recovery compared to the single-stage one [30].

These cost-effective eco-friendly biotechnologies are based on microbial activities with special consortia that lead to directly proportional production of the energy carriers hydrogen and methane. The coproduction of hydrogen and methane from corn stalk by a multi-stage anaerobic fermentation process was reported by Cheng et al. [31], while the byproduct of food industry molasses was used as a sole carbon source for the two-stage biogas-producing process in the studies of Park et al. [32]. The generation of the energy carriers biohydrogen and biomethane in two consecutive steps could be the most favorable since they could be generated continuously with high production rates utilizing organic waste that is generated from the food industry.

**Figure 4:**
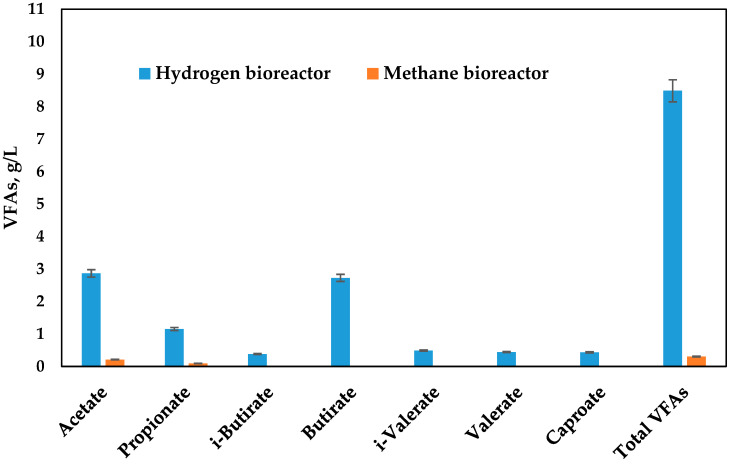
VFAs produced during dark fermentation in the hydrogen bioreactor and after methane production.

Volatile fatty acids are one of the liquid products, being very important as a cross point between the two bioreactors. They are a direct substrate for methanogenic microorganisms. In the performed process, their production is summarized in Figure 4. Acetate and butyrate are the most abundant VFAs present in the mixture, with 2.87 g/L (34% of total VFAs) content for acetate and 2.73 g/L (31% of total VFAs) for butyrate. The total VFAs content is about 8.49 g/L. The propionate is usually a non-desirable product because of hydrogen consumption during its production [9]. Moreover, it has a significant value but still about 2.5 times less in comparison with acetate and butyrate. When transferred with the liquid fraction to the second bioreactor, their concentration reaches up to 1.53 g/L, and further, they are being consumed up to 91% during methanogenesis. The acetate is the major VFA detected in the second bioreactor in a quantity 0.21 g/L, but it is probably because of its additional production in the second bioreactor. In the studies of other authors, the VFAs’ consumption is reported to be about 99.5–100% [33]; however, these data are provided by biochemical methane potential at batch studies with a duration of 30 days.

**Figure 5:**
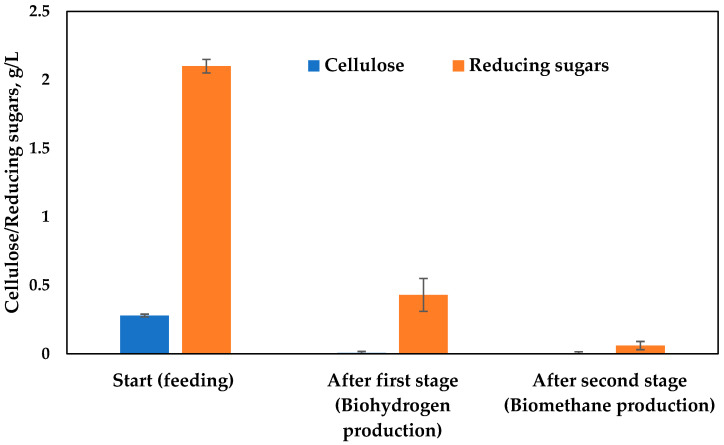
Reducing sugars and cellulose content.

During the bioreactors performances, cellulose and reducing sugars content were analyzed, showing their depletion at the expense of energy carriers production (Figure 5). The cellulose is present in small quantities in this type of substrate used at the start of the process and is fully degraded in the first bioreactor. The processes implemented in the two-phase system are continuous processes with daily feeding. Since the data are similar, the data presented in Figure 4 and Figure 5 are averaged data from measurements taken during the process.

The quantity of reduced sugars after the hydrolysis phase in the first bioreactor decreased 5-fold and was fully utilized in the second methanogenic phase (second bioreactor). The total reducing sugars consumed were 96.8% in a process of anaerobic digestion of hydrolyzed corncob waste, as reported by Sulbaran-Angel et al. [34].

The main results in this study concern the characterization of the complex microbial community inhabiting the two bioreactors of the anaerobic system with corn steep liquor as a substrate, and they shed light on the anaerobic digestion processes carried out by the different microbial groups.

Anaerobic digestion is a biochemical process with participants—four groups of microorganisms: hydrolyzers, acidogens, acetogens and methanogens—which play an important role and act synergistically in dependence of the metabolic pathways. The composition of the microbial communities in anaerobic bioreactors depends on the inoculum used, the type of substrate and the process conditions. 

The methods for pretreating the inoculum to start a hydrogen process are various. Among them, thermal treatment is one of the common methods of getting rid of methanogenic microorganisms. According to some authors, this type of treatment (where a range of temperature regimes from 70 °C to the boiling of the sample are offered) is suitable for the treatment of mesophilic inoculum and leads to the suppression of the development of hydrogen-consuming microorganisms. In addition to the thermal treatment, the lowered starting pH gives additional assurance that the development of methanogenic microorganisms will be hindered. This fact was confirmed in our results by the lack of methane production in the first hydrogen-generating bioreactor and was also proved by the metagenomics analyses, showing negligible quantities of archaea in the first bioreactor.

Microbial diversity is often accompanied by a vast metabolic capability reflected in precise consecutive biochemical processes. It is influenced by the factors of the surrounding environment that have their impact on the predominance of certain species or lack of others. So, by altering the favorable conditions for certain species, the balance could be changed, and the direction of the whole process could be altered. 

In our analyses of the biogas of the first hydrogenic reactor, no methanogens were found—only hydrolytic and hydrogen generating microorganisms are available, working synergistically and leading to a stable process, as seen from the hydrogen yield—while in the second methanogenic reactor, bacterial and archaeal methanogens were determined.

Knowledge about the composition and the changes in the microbial communities involved could help avoid process failures. The continuous feeding of the substrate leads to community shifts that contribute to a stable reactor performance. A longer starving period or a change in the pH value could result in further community shifts within one phylum but did not influence another. Microbial community dynamics during anaerobic digestion are essential to improving the process efficiency for increasing hydrogen and methane yield. In this study, bacterial, archaeal and fungal diversity were investigated and defined.

**Figure 6:**
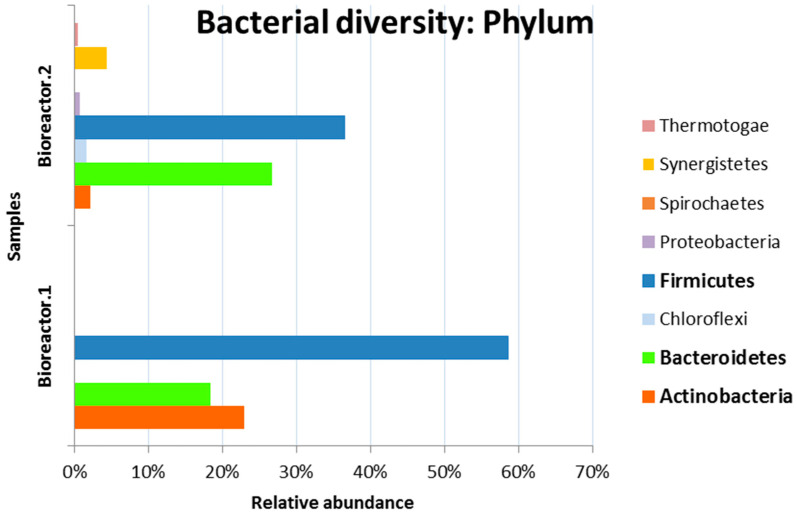
Phylum level bacterial taxonomy of Bioreactor.1 and Bioreactor.2 communities based on the average 16S amplicon datasets of each community.

The obtained metagenomic data showed that the most abundant Phylum in both bacterial communities was *Firmicutes*—58.61% and 36.49% in bioreactors 1 and 2, respectively (Figure 6).

The dominance of *Firmicutes* in biogas reactors was previously demonstrated by Klocke et al. [35]. *Actinobacteria* phylum was also found in significant amounts (22.91%) in the microbial community in Bioreactor 1, while in Bioreactor 2, they were 2.1%. Phylum *Bacteroidetes* were present in both bioreactors. Enhancement of biogas production and degradation efficiency by *Bacteroides*, participating in a methanogenic consortium, was reported by Tukanghan et al. [36]. As described by Kampnm et al. [37], the phyla *Bacteroidetes* and *Firmicutes* dominated, with 58.9% and 30.1% of the sequences, respectively, in processes conveyed in laboratory biogas reactors fed with different defined substrates containing liquid manure consecutively fed with casein, starch and cream over a period of up to 33 days.

Phylum *Euryarchaeota* from the kingdom *Archaea* was 0.4% in the first and 11.4% in the second bioreactor, with the dominant genera among methanogenic archaea being *Methanothrix* and *Methanosarcina*. Methanogenic archaea, or methanogens, have drawn considerable attention recently for their critical role in the global carbon cycle because of their unique ability to produce methane. *Methanothrix* species were reported to be the dominant and metabolically active methanogens in a methanogenic sludge fed with ethanol-type fermentation products [38]. At low concentrations of acetate, filamentous *Methanosaeta* species usually dominate. Normally, high concentrations of some toxic ionic agents, such as ammonia, hydrogen sulfide and volatile fatty acids, inhibit *Methanosaetaceae* and especially allow the growth of *Methanosarcina* species [9].

**Figure 7:**
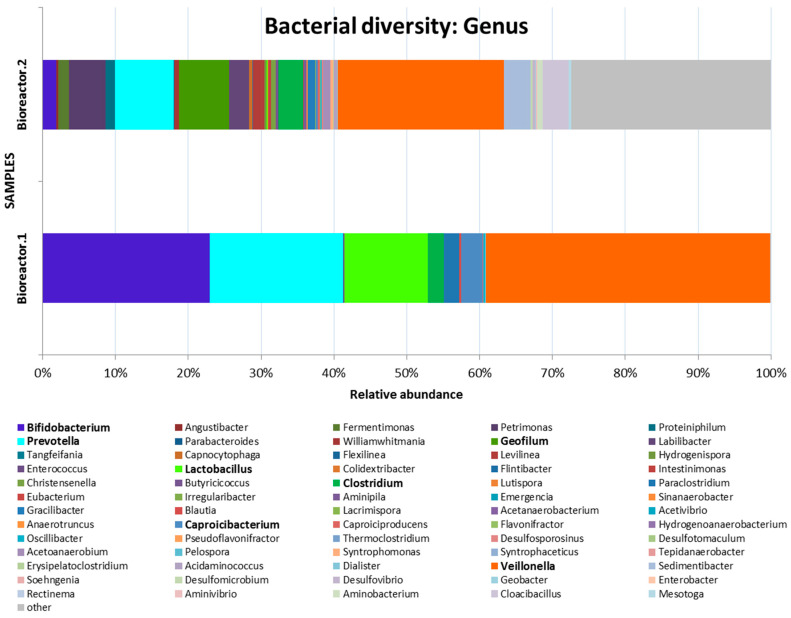
Genus level bacterial taxonomy of Bioreactor.1 and Bioreactor.2 communities based on the average 16S amplicon datasets of each community.

The bacteria in the first bioreactor were dominated by representatives of the genera *Veillonella* (39.00%), *Bifidobacterium* (22.91%), *Prevotella* (18.32%), *Lactobacillus* (11.48%) and *Caproicibacterium* (2.92%). The second bioreactor mainly contained *Veillonella* (22.77%), *Prevotella* (8.05%), *Geofilum* (6.84%), *Petrimonas* (4.98%) and *Sedimentibacter* (3.65%), as presented in Figure 7.

**Figure 8:**
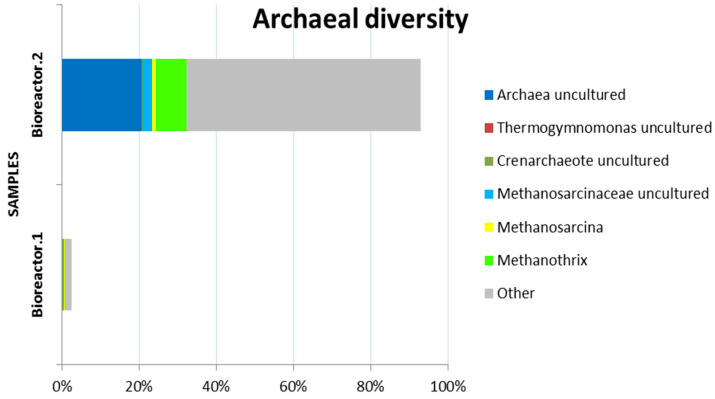
Relative abundance of archaea phyla of Bioreactor.1 and Bioreactor.2 in two-phase anaerobic biodegradation with corn steep liquor.

The day (sample taking) corresponding to the data displayed in Figure 6, Figure 7 and Figure 8 was the one during which the whole volume of the second methanogenic bioreactor was totally exchanged for the purpose of reaching a stable microbial communty.

The presence of a significant amount of lactobacilli in the first reactor is most likely due to the use of CSL as a substrate. CSL is a good source of organic nitrogen and contains sugars, amino acids, vitamins, minerals and microorganisms, mainly lactobacilli [35,36]. *Lactobacillus* species in the first bioreactor metabolize hexoses to lactate, the main fermentation end product. *Caproicibacterium amylolyticum* also produces lactate. The representatives of the genus *Veillonella* are anaerobic bacteria capable of fermenting lactate and, as can be seen, they are significantly (39.00%) represented in the first bioreactor. 

The great amount of anaerobic bacteria of the genus *Bifidobacterium* in the first hydrogenic bioreactor is probably due to their ability to degrade plant-derived fructo-oligosaccharides available in the CSL medium. The defined microbial community data differ significantly from some other similar studies, mainly due to the difference in the substrate used [39,40]. According to Kabaivanova et al. [41], biohydrogen generation is most probably due to the presence of *Proteiniphilum saccharofermentans*, proved to be 28.2% to 45.4% of the microbial community in the first and the second bioreactor, respectively, when wheat straw was used as a substrate. Seon et al. found that *Bacteroides*- and *Clostridium*-related microorganisms are responsible for the hydrolysis of alginate and the production of volatile fatty acids [42]. 

The use of different substrates results in different C/N ratios. The balance of the C/N ratio has a major influence on the bacterial composition. The same ratio affects not only the population structures of bacteria and archaea, but also microbial community functions and biogas production [43,44]. The better performance of the reactor may be due to the predominance of *Firmicutes* and the greater bacterial diversity. *Firmicutes* and *Chloroflexi* are known to be able to degrade a large number of organic compounds under various conditions [45]. These bacterial species have tolerance to complex organic loading. Bacteria are responsible for breaking down substrates into intermediate metabolites that can later be used by methanogens. There are mainly three types of methanogens, namely, acetoclastic, hydrogenotrophic and methylotrophic. Most of the CH_4_ is produced by the first two types [46].

In this study, *Archaea* were predominantly detected in the second reactor, belonging to the family *Methanotrichaceae* (8.03%) and *Methanosarcinaceae* (3.39%), with the analysis reporting about 20% of unclassified archaea (Figure 8). 

The genus *Methanosaeta* and *Methanothrix* belong to the family *Methanotrichaceae* and they are acetoclastic methanogens. The representatives of the family *Methanosarcinaceae* are methanogens that incorporate the unusual amino acid pyrrolysine into their enzymes [47]. The enzyme monomethylamine methyltransferase catalyzes the reaction of monomethylamine to methane [48]. The species of genus *Methanosarcina* may be the only known anaerobic methanogens that produce methane using all three known metabolic pathways for methanogenesis [49]. These genera are probably responsible for the production of methane in the second bioreactor of the system and were identified through the metagenome analysis performed in this study.

In both reactors, fungi are present with representatives of the species *Saccharomyces cerevisiae* (first reactor 98.65%) and (second reactor 96.18%), probably due to the type of substrate used. On one hand, *Saccharomyces cerevisiae* is a yeast which can successfully grow in the absence of oxygen; on the other, the effect of adding yeast on biogas production performance, when the substrate is added after biogas production, is reduced, as has been demonstrates by Ming et al. [50]. 

However, in both bioreactors, the bacterial diversity is most pronounced, with different groups of bacteria predominating.

Anaerobic digestion, mediated by novel microbial consortia, is widely used to convert different wastes to green energy. While the main microorganisms and mechanisms involved in the methane-producing anaerobic microbial cell factories are fairly well known, the management and regulation of the overall process is, as yet, far from being totally understood. Comprehensive and predictive elucidation of the exact composition of microbial consortia participating in the consecutive biochemical conversions of the process will assist in coping with this research challenge.

## 4. Conclusions

In this study, the bacterial, archaeal and fungal communities were identified using metagenomics for the first time in a two-stage system of anaerobic bioreactors for hydrogen and methane production from corn steep liquor. The phylum prevailing in both bacterial communities was *Firmicutes* in the hydrogenic and methanogenic bioreactors, followed by *Bacteroidetes*. During the bioreactors performances, cellulose and reducing sugars depletion was monitored at the expense of energy carriers production. The volatile fatty acids, as one of the liquid products and an important cross point between the two bioreactors, were measured, with acetate and butyrate in the highest quantities. 

Knowledge of the composition and network interaction between different members of the microbial community can contribute to process optimization and production enhancement to avoid process failure.

The production of biogas using newly identified anaerobic microbial pollutions with waste substrate from food industry is an important step towards a more sustainable and resource-efficient future and can help address a range of environmental issues.

## Figures and Tables

**Table 1:** ADM^®^ Corn Steep Liquor 104 characteristics.

Parameter	Value
pH	4.2 ± 0.1
TS, %	50.5 ± 0.2
VS, %	91 ± 0.3
Reducing sugars, g/L	70.6 ± 2.4
Cellulose, g/L	2.46 ± 0.1

## Data Availability

Not applicable.
